# Knowledge, attitudes, and practices of adolescents regarding human papillomavirus

**DOI:** 10.11606/s1518-8787.2022056003639

**Published:** 2022-03-23

**Authors:** Mariana Portela Soares Pires Galvão, Telma Maria Evangelista de Araújo, Silvana Santiago da Rocha

**Affiliations:** I Fundação Municipal de Saúde de Teresina Teresina PI Brasil Fundação Municipal de Saúde de Teresina. Teresina, PI, Brasil; II Universidade Federal do Piauí Centro de Ciências da Saúde Departamento de Enfermagem Teresina PI Brasil Universidade Federal do Piauí. Centro de Ciências da Saúde. Departamento de Enfermagem. Teresina, PI, Brasil

**Keywords:** Adolescent, Papillomavirus Infections, prevention & control, Papillomavirus Vaccines, Health Knowledge, Attitudes, Practice

## Abstract

**OBJECTIVE:**

To analyze knowledge, attitudes, and practices of adolescent students from public schools in the municipality of Teresina, state of Piauí, regarding human papillomavirus (HPV).

**METHODS:**

Cross-sectional, analytical study carried out in 12 public schools in the municipality of Teresina, with a random sample of 472 15-year-old adolescents. All participants answered a validated questionnaire, which evaluated sociodemographic characteristics, level of knowledge about HPV, attitudes regarding vaccination and vaccination status. The levels of knowledge and attitude were classified by standardized scores and practice by the vaccination situation. The analyses were carried out using the SPSS software. In the bivariate analysis, simple logistic regression was used generating odds ratios to identify the associations of sociodemographic characteristics, knowledge, and attitude with HPV prevention practice. Variables that presented p-value ≤ 0.20 in the bivariate analysis were inserted in a multivariate logistic regression model. Statistical significance was set at p < 0.05.

**RESULTS:**

Among the participants, 27.3% had sufficient knowledge, 34.1% had positive attitudes, and 74.6% had adequate practice. In the multivariate analysis, we observed a statistically significant association among females (ORa = 15.62; 95%CI: 9.08–26.9), satisfactory knowledge (ORa = 2.09; 95%CI: 1.15–3.81), and positive attitudes (ORa = 1.89; 95%CI: 1.10–3.23) with proper practice.

**CONCLUSIONS:**

Being female, having a satisfactory level of knowledge about HPV and the vaccine, and having positive attitudes towards HPV vaccination reinforce the appropriate practice of vaccination. These findings demonstrate the need to expand the knowledge of adolescents, generating positive attitudes towards vaccination within an appropriate perspective.

## INTRODUCTION

Human papillomavirus (HPV) infection is extremely frequent worldwide and its transmission is mainly due to sexual contact, estimations point to 80% of sexually active individuals being infected with HPV at some point in their lives^1^. In Brazil, preliminary data from a 2017 population-based study conducted in 26 Brazilian capitals and the Federal District identified an HPV infection prevalence in 54.6% of the collected samples, of these, 38.3% had high-risk HPV. In the municipality of Teresina, state of Piauí, the detected prevalence was 53.3%^2^.

Although most times the infection is transient, its persistence has been directly associated with the development of cervical cancer, which represents an important health problem nowadays, due to its high incidence^3^. In Brazil, cervical cancer is the third most frequent type of cancer among women, with higher incidences recorded in states with lower socioeconomic development, such as those of the North and Northeast^4^.

Vaccination and cytological examination represent the main strategies for HPV prevention^5^. Immunization has shown important results in reducing the rates infection by the virus in countries where vaccination coverage is high and already has a proven impact on reducing the incidence of cervical cancer^1,6^. However, maintaining high vaccination coverage has been a challenge in Brazil^5^.

In this context, adolescents, who are the target audience of immunization, must have knowledge and awareness about the virus and recognize the importance of vaccination^5^. Also, note that currently adolescents constitute a group of high vulnerability to sexually transmitted infections (STIs) such as HPV, due to the early development of sexuality, the multiplicity of partners, greater sexual freedom, need for group affirmation, allied to resistance to condom use^7^.

Thus, considering the importance of HPV vaccination and the high risk of exposure of adolescents to the virus, the need for investigation on the diagnostic conjuncture about what adolescents know, think, and practice regarding this health problem emerged. Thus, this study aimed to analyze the knowledge, attitudes, and practices of adolescent students from public schools in the municipality of Teresina, Piauí, regarding HPV.

## METHOD

Cross-sectional, analytical study, developed by using a knowledge, attitude, and practice (KAP) survey, conducted in public high schools in the municipality of Teresina, capital of Piauí, whose population, according to the Brazilian Institute of Geography and Statistics (IBGE)^8^, is 850,198 inhabitants.

The management of the state public schools of Teresina is organized in four Regional Teaching Management units (GRE). The inclusion criteria for the selection of schools in the study were: in the urban area of the capital and offering regular or comprehensive high school. Among 139 public schools, 91 met this inclusion criterion, distributed as follows: 26 in the 4th GRE; 25 on the 19th GRE; 18 on the 20th GRE; and 22 in the 21st GRE.

For the study population, the inclusion criterion was to be a 15-year-old adolescent, enrolled in high school and regularly attending the selected schools at the time of collection. The choice for this age group occurred because by the age of 15, according to the National Immunization Program (PNI), the adolescent must have already received both doses of the HPV vaccine.

To calculate the sample of students, a proportional stratified probabilistic sample was used, considering the population of 10,923 15-year-old adolescents of both sexes, enrolled in public high school, attending the 1st grade in the 2018 school year.

The sample size was calculated by random sample for proportions, adopting a 95% confidence interval (95%CI), prevalence of 29% (considering the study by Osis, Duarte and Sousa^9^, which identified 28.9% of the population with adequate knowledge about HPV), accuracy of 5%, and significance level of 5%, obtaining a minimum sample of 386 adolescents. Applying a 20% rate for sample recomposition and assuming possible loss during the study, the planned sample size was at least 463 participants.

Three schools were drawn for each geographic area, totaling 12 schools, since this number was considered sufficient to obtain the number of adolescents needed for the planned sample. The distribution of the sample in the selected schools was proportional to the number of students. After this first proportional distribution, the disposition was defined by the number of classes, then the proportional distribution according to the sex of the students, using the Software R, version 3.4.0.

Data collection occurred from October to December 2018 and an instrument previously validated in English, in 2012^10^, was used to measure the level of knowledge about HPV and attitudes regarding vaccination. The instrument was adapted for Portuguese in Brazil in 2016^11^and was used after prior authorization by the author.

The variables of interest analyzed were: sociodemographic (gender and race/color) and economic characteristics and evaluation of knowledge, attitude, and practice. The economic characteristics of the families of adolescents were represented by an indicator constructed from information on quality of life, related to the health of the adolescents^12^ and the synthesis of social indicators^13^, called ‘indicator of possessions (IP).’

The ‘indicator of possessions (IP)’ was calculated by: IP = ∑ (1 − *ƒ*i) bii. Where: i ranges from 1 to 7 goods; bi is equal to 1 or zero respectively, in the presence or absence of landline, cell phone, internet, automobile, motorcycle, computer (desktop, notebook etc.), bathroom with shower. The weight attributed to the presence of each household good will be complementary to the relative frequency (*ƒ*i) of each good in the total sample, that is, the rarer the good in the household, the greater the weight attributed to it.

The indicator was further refined by adding a weight that considers the amount of the “i” good found in the household (and not only whether it exists in the household). This variant is calculated by w = 0 to k, where “k” indicates the amount of the “i” good in the household. The dichotomized form of the variable was used, initially in quintiles, to separate the subgroup with the lowest possession of goods – supposedly the “least economically favored” – from the group with the highest possessions^12^.

The evaluation of knowledge, attitude, and practice was consolidated into three categories: I disagree (I totally disagree and I do not agree), I do not agree nor disagree, or I agree (I agree and I strongly agree); and it was conducted as follows: to classify the degree of knowledge of adolescents regarding HPV, the answers of the questionnaire on the subject were evaluated with a numerical value assigned for each answer and the correct answers totaling six points, corresponding to 100%.

Subsequently, scores adapted from the study by Almeida et al.^14^, in which the degree of knowledge was categorized as unsatisfactory knowledge (0 to 74%) and satisfactory knowledge (75 to 100%). The answers were considered adequately correct when: the adolescent disagreed that men do not get HPV, agreed that women vaccinated against HPV need to undergo Pap tests, agreed that HPV can be contracted through sexual activity, disagreed that HPV is very rare, agreed that HPV can cause cervical cancer, and disagreed that the HPV vaccine protects against all types of cancer.

Regarding the determination of attitudes, the instrument was composed of positive and negative statements about the aspects involving vaccination and each alternative was assigned a numerical value, with the most positive attitude totaling eight points, which corresponds to 100%. Subsequently, the attitude was classified according to the percentage as follows: from 0 to 74% negative attitude, from 75 to 100% positive attitude.

The attitude was considered positive when the adolescent agreed that he strongly values his health, agreed that the prevention of diseases and infections is important to him, disagreed that a vaccine shot can be a bother, disagreed that he fears that HPV vaccines are very painful, agreed that he is not afraid to receive vaccines, disagreed that he is concerned about the side effects of the vaccine, disagreed that he feels tense when he hears other boys talking about HPV vaccination, agreed that he is concerned that he may develop HPV cancer in the future.

The practice was evaluated using the vaccination schedule. The adolescent responding that he has received both doses of the HPV vaccine was considered an appropriate practice.

All variables of the instrument for data collection were organized and coded in a dictionary called codebook. Then, this data was entered in a Microsoft Office Excel 2016 spreadsheet, and after double typing, the data was exported to the Statistical Package for the Social Sciences (SPSS), version 21, to perform the statistical analyses. Adolescents whose questionnaires were incomplete were excluded.

Sociodemographic data and the classification of knowledge, attitude, and practice (vaccination situation) were initially analyzed by descriptive statistics. The bivariate test of association between qualitative variables (sociodemographic, knowledge, and attitude) used was the simple logistic regression, referred to here as unadjusted odds, to select the possible variables that could explain the appropriate practice.

To explain the joint effect of the variables on the appropriate practical outcome (yes/no) multiple logistic regression was used using the adjusted Odds Ratio (ORa). The inclusion criterion of variables in the logistic model was the p < 0.20 level association in the bivariate analysis^15^. However, the significance criterion of the variables in the model was the p < 0.05 level association.

This study was approved by the Research Ethics Committee of the Universidade Federal do Piauí under Opinion No. 2,868,990, in accordance with Resolution No. 466/2012. The adolescents signed an informed assent form, and the parents or guardians signed an informed consent form as a form of common agreement to participate in the research.

## RESULTS

A total of 472 students participated in the study since, at the time of data collection, all adolescents aged 15 years in one of the selected classrooms expressed the desire to participate. Of the total, 60.8% were female, 39.8% were male and most considered themselves as mixed race (60.8%). The evaluation of the economic factor resulted in dichotomous variables, having as cut-off point the quintiles, in which the group with the lowest possession of goods scored up to the 1st quintile and the group with the highest possession of goods scored above the 1st quintile. Most adolescents (79.7%) belonged to the group with the highest possession of goods (Table 1).

**Table 1:** Sociodemographic characterization of the study teenager participants. Teresina, Piauí, Brazil, 2019 (n = 472).

Variables	n	%
Gender		
Male	185	39.2
Female	287	60.8
Ethnicity		
White	68	14.4
Black	65	13.8
Mixed race	287	60.8
Others (Asian or indigenous)	52	11.0
Indicator of possessions		
Group with least possession of goods (up to the 1st quintile)	96	20.3
Group with more possession of goods (over the 1st quintile)	376	79.7

The percentage of adolescents with insufficient knowledge about HPV was 72.7%, and 65.9% had negative attitudes towards infection prevention. We identified the appropriate practice (being vaccinated) in 74.6% of adolescents (Figure).

**Figure:**
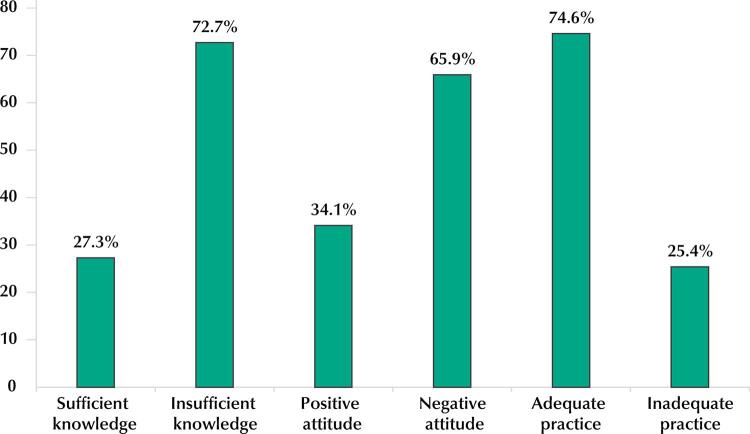
Knowledge, attitudes, and practices of the study sample regarding HPV. Teresina, Piauí, Brazil, 2019.

Females are more vaccinated (92.7%) than males (46.5%). The race/color variable showed no significant differences between the categories regarding the practice. The evaluation of the variable indicator of possessions identified a slightly higher percentage of vaccinated adolescents (77.1%) in the group with the lowest possession of goods (Table 2).

**Table 2:** Association between sociodemographic variables, knowledge, and attitude with HPV vaccination practice. Teresina, Piauí, Brazil, 2019.

Variables	Practice (vaccinated against HPV virus)		Crude Odds (95%CI)	p

	Yes	No		
Gender				
Female	266 (92.7%)	21 (7.3%)	14.58 (8.58–24.77)	< 0.001
Male	86 (46.5%)	99 (53.5%)	1	
Race/color				
White	52 (76.5%)	16 (23.5%)	0.97 (0.41–2.29)	0.954
Black	46 (70.8%)	19 (29.2%)	0.72 (0.31–1.67)	0.454
Mixed race	214 (74.6%)	73 (25.4%)	0.87 (0.43–1.76)	0.718
Asian or indigenous	40 (76.9%)	12 (23.1%)	1	
Indicator of possessions				
Group with least possession of goods (up to the 1st quintile)	74 (77.1%)	22 (22.9%)	1.18 (0.69–2.01)	0.527
Group with more possession of goods (over the 1st quintile)	278 (73.9%)	98 (26.1%)	1	
Knowledge				
Sufficient	108 (83.7%)	21 (16.3%)	2.08 (1.23–3.51)	0.005
Insufficient	244 (71.1%)	99 (28.9%)	1	
Attitude				
Positive	128 (79.5%)	33 (20.5%)	1.50 (0.95–2.37)	0.077
Negative	224 (72.0%)	87 (28.0%)	1	

ORc: Crude odds ratio; 95%CI: 95% confidence interval; p-value: value of p.

When assessing the association between sociodemographic variables, knowledge, and attitude with practice, we observed that the female gender (ORc = 14.58; 95%CI: 8.58–24.77) and knowledge (ORc = 2.08; 95%CI: 1.23–3.51) were statistically associated (Table 2).

In the multivariate model, the variables that explained the proper practice were sex, knowledge, and attitude. Female adolescents are 15.62 times more likely to be vaccinated (ORa = 15.62; 95%CI: 9.08–26.90), sufficient knowledge increases this chance by 2.09 times (ORa = 2.09; 95%CI: 1.15–3.81) and positive attitude increases vaccination by 1.89 times (ORa = 1.89; 95%CI: 1.10–3.23) (Table 3).

**Table 3:** Logistic regression of variables associated with appropriate practice against HPV. Teresina, Piauí, Brazil, 2019.

Variables	Vaccinated against HPV virus		ORc (95%CI)	p	ORa (95%CI)	p

	Yes n (%)	No n (%)				
Gender						
Female	266 (92.7%)	21 (7.3%)	14.58 (8.58–24.77)	< 0.001	15.62 (9.08–26.90)	< 0.001
Male	86 (46.5%)	99 (53.5%)	1		1	
Knowledge						
Sufficient	108 (83.7%)	21 (16.3%)	2.08(1.23–3.51)	0.005	2.09 (1.15–3.81)	0.015
Insufficient	244 (71.1%)	99 (28.9%)	1		1	
Attitude						
Positive	128 (79.5%)	33 (20.5%)	1.50 (0.95–2.37)	0.077	1.89 (1.10–3.23)	0.019
Negative	224 (72.0%)	87 (28.0%)	1		1	

ORc: Crude odds ratio; 95%CI: 95% confidence interval; p-value: value of p; ORa: Adjusted odds ratio.

## DISCUSSION

Most adolescents participating in this study are female, declare themselves as mixed race and fall into the group with the highest possession of property. The profile data of this sample regarding gender and race/color are close to the analysis of the National School Health Survey 2015, conducted with a national sample of 102,301 9th graders, in which most, 51.3%, are female, and mixed race, 43.1%^16^. A study conducted in Rio de Janeiro, municipalities of Niterói and São Gonçalo, in 2018, with 807 adolescents, also included most participants (80.3%) in the group with the highest possession of goods, despite covering students from public and private schools^12^.

Although knowledge about HPV plays a crucial role in how people assume their sexuality, protection, and prevention^17^, the results of this research showed that most adolescents had insufficient knowledge about this condition. This corroborates other publications that point to insufficient knowledge about HPV among adolescent^18^ and in different population groups^19^. In Brazil, the study with national sampling^5^ conducted with young people aged 16 to 25 years identified an average percentage of 51.79% of correct answers regarding HPV and vaccination. In Mexico^17^, a survey with 242 students between 14 and 18 years old reported that 80% of them had a low level of knowledge about HPV.

We observed that the female public presented a higher level of knowledge than the male, similar to the findings of other studies^19,20^. However, when considering the level of knowledge only among the female public, we found a high percentage (70.7%) with insufficient knowledge, as in another national study^19^, which, despite the larger percentage of women than that of men, observed a low proportion of well-informed individuals.

Most adolescents presented negative attitudes towards HPV prevention and vaccination. We evaluated aspects related to the perception of the importance of prevention and the fear and discomfort when receiving the vaccine. The perception of the severity and susceptibility of the individual to HPV infection, and their beliefs about the benefits and barriers to vaccination can predict the intention to receive the vaccine, and this intention predicts behavior^21^.

The attitudes and beliefs of parents and adolescents regarding the safety and efficacy of the vaccine, concerns about side effects, lack of conviction that the vaccine is essential, especially among males, and the lack of awareness about HPV infection and its associated risks, are factors that hinder the maintenance of high vaccination coverage^22^.

At the beginning of the quadrivalent HPV vaccine implementation in the Unified Health System (SUS), which occurred in 2014, the country achieved a high vaccination coverage in the first phase of the campaign; however, the second dose showed a significant and progressive reduction^23^that resulted in only 60.15% of vaccinated adolescents^24^. This reduction is assumed to be a result of the first dose being administered in public and private schools, while the second dose was available in the Basic Health Units, like the current doses are, and few health professionals went to the target audience to administer the second dose in schools^23^. In the year following the vaccine implementation coverage decreased substantially^25^. This picture shows that the initial vaccination strategy in schools adopted in Brazil showed more success in the adhesion rates.

The results showed that females had much higher chances of getting vaccinated when compared with males. The vaccine being initially made available by the SUS only for females and the dissemination of information regarding the consequences of HPV in women, such as cervical cancer, possibly contributes to the ignorance of the availability of this vaccination for both sexes and for the lower vaccination coverage among male adolescents^26^.

In this study, no sociodemographic characteristic was associated with knowledge and attitude about HPV. However, in another study conducted in Brazil, the Black race was associated with low levels of knowledge about HPV among women^27^. In the United States, a study showed that Black women were less likely to have knowledge about the HPV vaccine compared to white women^28^. This scenario is a likely result of the greater access to the vaccine and to knowledge on the subject among white people, influenced by socioeconomic factors^29^.

Females, sufficient knowledge, and positive attitudes about the vaccine and HPV were associated with adequate prevention practice by vaccination. However, the low rate of adolescents with sufficient knowledge and positive attitudes is a worrying scenario since it negatively influences acceptability and vaccine adhesion, as demonstrated in our study and other publications^29,30^, such as a study with high school students in Malaysia^30^, which identified that the intent to get a vaccination was significantly associated with the level of knowledge about cervical cancer. This scenario highlights the need for educational interventions aimed at raising the awareness of the population about the problem of the infection and the need for preventive measures.

The main strength of our study was to investigate with a sample that covered all public schools in the urban area of the municipality of Teresina, which provided an overview of the knowledge and attitudes of these students regarding the subject and allowed an evaluation of the rates of HPV vaccine adhesion. Another strong point of this study was to cover adolescents who recently became part of the target audience of HPV immunization in the public health network.

Among the limitations is the lack of a standardized scale for the evaluation of knowledge and attitudes about the subject, we made the choice for the scoring procedure due to finding it more appropriate than evaluating with isolated questions. However, we consider that this study could provide important contributions to health strategies and policies aimed at the intensification of HPV vaccine coverage. The occurrence of information bias is also possible considering the self-declared answers, especially those related to sexual questions, which could lead adolescents to change their answers for fear of moral judgments. We used instruments with self-response to minimize those.

## CONCLUSION

The findings of this study demonstrated significant associations between sufficient knowledge and positive attitudes with the practice of vaccination, which shows that expanding the knowledge of adolescents, generating favorable attitudes, can be a valuable tool for HPV vaccine adhesion.

The results also showed a low rate of vaccinated male adolescents and an association between females and HPV vaccination. This scenario indicates the need to include the male population in themes inherent to HPV prevention since infection is often associated only with cervical cancer, which underestimates its consequences among men, contributing to low vaccination coverage in this public.
