# Supplementary material for: Knowledge, attitudes, and practices of adolescents regarding human papillomavirus
Source: Rev Saude Publica. 2022 Mar 23;56:12. doi: 10.11606/s1518-8787.2022056003639 (PMC8963747; doi:10.11606/s1518-8787.2022056003639)
Supplement: Translation

# Translation: Conhecimentos, atitudes e práticas de adolescentes sobre o papilomavírus humano

**Authors:** Mariana Portela Soares Pires Galvão, Telma Maria Evangelista de Araújo, Silvana Santiago da Rocha

**Affiliations:** I Brasil Fundação Municipal de Saúde de Teresina. Teresina, PI, Brasil; II Brasil Universidade Federal do Piauí. Centro de Ciências da Saúde. Departamento de Enfermagem. Teresina, PI, Brasil

**Keywords:** Adolescente, Infecções por Papillomavirus, prevenção & controle, Vacinas contra Papillomavirus, Conhecimentos, Atitudes e Prática em Saúde

## Abstract

**OBJETIVO:**

Analisar conhecimentos, atitudes e práticas de adolescentes estudantes de escolas públicas do município de Teresina-PI sobre o papilomavírus humano (HPV).

**MÉTODOS:**

Estudo transversal, analítico, realizado em 12 escolas da rede pública do município de Teresina, com uma amostra aleatória de 472 adolescentes de 15 anos. Todos os participantes responderam a um questionário validado, que avaliou as características sociodemográficas, o nível de conhecimento sobre o HPV, atitudes relacionadas à vacinação e ao status vacinal. Os níveis de conhecimento e atitude foram classificados por meio de escores padronizados e a prática mediante a situação vacinal. As análises foram realizadas com o uso do SPSS. Na análise bivariada, utilizou-se a regressão logística simples, por meio de *odds ratio* para identificar as associações entre as características sociodemográficas e o conhecimento, e a atitude com a prática de prevenção contra o HPV. As variáveis que na análise bivariada apresentaram valor de p ≤ 0,20 foram submetidas ao modelo multivariado de regressão logística. A significância estatística foi fixada em p < 0,05.

**RESULTADOS:**

Dentre os participantes, 27,3% apresentaram conhecimento suficiente, 34,1% atitudes positivas e 74,6% prática adequada. Na análise multivariada observou-se associação estatisticamente significativa entre o sexo feminino (ORa = 15,62; IC95%: 9,08–26,9), conhecimento satisfatório (ORa = 2,09; IC95%: 1,15–3,81), e atitudes positivas (ORa = 1,89; IC95%: 1,10–3,23) com a prática adequada.

**CONCLUSÕES:**

Ser do sexo feminino, ter nível de conhecimento sobre o HPV e a vacina classificados como satisfatório, bem como ter atitudes positivas frente à vacinação contra o HPV reforçam a prática adequada de vacinação. Estes achados demonstram a necessidade de ampliar o conhecimento dos adolescentes, gerando atitudes positivas com vistas à vacinação dentro de uma perspectiva adequada.

## INTRODUÇÃO

A infecção pelo papilomavírus humano (HPV) é extremamente frequente em todo o mundo e sua transmissão se dá principalmente pelo contato sexual, estima-se que 80% dos indivíduos sexualmente ativos serão infectados pelo HPV em algum momento da vida^1^. No Brasil, dados preliminares de um estudo de base populacional, de 2017, realizado em 26 capitais brasileiras e no Distrito Federal, identificaram uma prevalência de infecção pelo HPV em 54,6% das amostras coletadas, dessas amostras, 38,3% apresentavam o HPV de alto risco. No município de Teresina-PI foi detectada uma prevalência de 53,3%^2^.

Embora na maioria das vezes a infecção seja transitória, sua persistência tem sido diretamente associada ao desenvolvimento de câncer do colo do útero, que representa um importante problema de saúde na atualidade, devido sua elevada incidência^3^. No Brasil, o câncer do colo do útero é o terceiro tipo de câncer mais frequente entre mulheres, com maiores incidências registradas em estados com menor nível de desenvolvimento socioeconômico, como o Norte e o Nordeste^4^.

A vacinação e o exame citológico representam as principais estratégias de prevenção contra o HPV^5^. A imunização mostrou resultados importantes na redução das taxas de infecções provocadas pelo vírus em países onde a cobertura vacinal é alta e já possui impacto comprovado na redução da incidência de câncer do colo do útero^1,6^. No entanto, a manutenção de elevadas coberturas vacinais tem sido um desafio no Brasil^5^.

Nesse contexto, é essencial que os adolescentes, que representam o público-alvo da imunização, tenham conhecimento e consciência sobre o vírus e reconheçam a importância da vacinação^5^. Além disso, destaca-se que atualmente os adolescentes constituem um grupo de alta vulnerabilidade às infecções sexualmente transmissíveis (IST) como HPV, devido ao desenvolvimento precoce da sexualidade, a multiplicidade de parceiros, maior liberdade sexual, necessidade de afirmação grupal, aliado à resistência ao uso de preservativo^7^.

Assim, considerando a importância da vacinação contra o HPV e o elevado risco de exposição dos adolescentes ao vírus, emergiu a necessidade de investigação a respeito da conjuntura diagnóstica sobre o que os adolescentes sabem, pensam e praticam frente a esse agravo à saúde. Dessa forma, o objetivo deste estudo foi analisar os conhecimentos, as atitudes e as práticas de adolescentes estudantes de escolas públicas do município de Teresina-PI sobre o HPV.

## MÉTODO

Estudo transversal, analítico, desenvolvido por meio de um inquérito de conhecimento, atitude e prática (CAP), realizado em escolas da rede pública que oferecem ensino médio no município de Teresina, capital do Piauí, cuja população, de acordo com o Instituto Brasileiro de Geografia e Estatística (IBGE)^8^, é de 850.198 habitantes.

A gestão das escolas públicas estaduais de Teresina está organizada em quatro Gerências Regionais de Ensino (GRE). Os critérios de inclusão utilizados para a seleção das escolas no estudo foram: estar localizada na zona urbana da capital e oferecer ensino médio regular ou integral. Dentre as 139 escolas públicas existentes, 91 atenderam a esse critério de inclusão, distribuídas da seguinte forma: 26 na 4ª GRE; 25 na 19ª; 18 na 20ª e 22 na 21ª GRE.

Para a população do estudo, adotou-se como critério de inclusão ser adolescente de 15 anos de idade, matriculado no ensino médio e frequentando regularmente as escolas selecionadas para o estudo no momento da coleta. A opção por esta faixa etária ocorreu porque ao completar 15 anos de idade, de acordo com o Programa Nacional de Imunizações (PNI), o adolescente já deve ter recebido as duas doses da vacina contra o HPV.

Para o cálculo da amostra de estudantes utilizou-se amostragem do tipo probabilística estratificada proporcional, considerando-se a população de 10.923 adolescentes de 15 anos, de ambos os sexos, matriculados no ensino médio público, cursando a 1ª série, no ano letivo de 2018.

O tamanho amostral foi calculado por meio de amostra aleatória para proporções, adotando-se intervalo de 95% de confiança (IC95%), prevalência de 29% (considerando-se o estudo de Osis, Duarte e Sousa^9^, que identificou 28,9% da população com conhecimento adequado sobre o HPV), precisão de 5% e nível de significância de 5%, obtendo-se amostra mínima exigida de 386 adolescentes. Aplicando-se uma taxa de 20% para recomposição da amostra e presumindo-se eventual perda durante a pesquisa, o tamanho amostral planejado foi de no mínimo 463 participantes.

Foram sorteadas três escolas para cada área geográfica, perfazendo o total de 12 escolas, tendo em vista que esse número foi considerado suficiente para se obter o quantitativo de adolescentes necessário para contemplar a amostra planejada. A distribuição da amostra nas escolas sorteadas foi proporcional ao número de alunos existentes. Após essa primeira distribuição proporcional, a disposição foi definida pelo número de turmas; seguida da distribuição proporcional segundo o sexo dos alunos, com a utilização do software R, versão 3.4.0.

A coleta de dados ocorreu no período de outubro a dezembro de 2018 e utilizou-se um instrumento previamente validado em inglês, em 2012^10^, com o objetivo de medir o nível de conhecimento sobre o HPV e atitudes relacionadas à vacinação. Contudo, o instrumento teve versão adaptada para o português no Brasil, em 2016^11^, e foi utilizada após autorização prévia da autora.

As variáveis de interesse analisadas foram: características sociodemográficas (sexo e raça/cor) e econômicas e avaliação do conhecimento, atitude e prática. As características econômicas das famílias dos adolescentes foram representadas por meio de um indicador construído a partir de informações sobre qualidade de vida, relacionada à saúde de adolescentes escolares^12^ e da síntese de indicadores sociais^13^, denominado ‘Indicador de Bens (IB)’.

O ‘Indicador de Bens (IB)’ foi calculado por: IB= ∑ (1-ƒi) bii. Onde: i varia de 1 até 7 bens; *bi* é igual a 1 ou zero respectivamente, na presença ou ausência de telefone fixo, telefone celular, internet, automóvel, moto, computador (de mesa, notebook etc.), banheiro com chuveiro. O peso atribuído à presença de cada bem do domicílio será complementar da frequência relativa (ƒi) de cada bem na amostra total, isto é, tanto mais rara a presença do bem no domicílio, tanto maior o peso atribuído ao bem.

O indicador foi ainda refinado adicionando um ponderador que considera a quantidade do bem “i” encontrada no domicílio (e não somente se este existe ou não no domicílio). Essa variante é calculada por w = 0 a k, em que “k” indica a quantidade do bem “i” no domicílio. Foi utilizada a forma dicotomizada da variável, inicialmente em quintis, para separar o subgrupo de menor posse de bens – pressupostamente o “menos favorecido economicamente” – do grupo com maior posse^12^.

A avaliação do conhecimento, atitude e prática foi consolidada em três categorias: discordo (discordo totalmente e não concordo), nem discordo nem concordo e concordo (concordo e concordo fortemente); e foi conduzida da seguinte forma: para classificar o grau de conhecimento dos adolescentes em relação ao HPV foram avaliadas as respostas do questionário sobre o tema, em que foi atribuído um valor numérico para cada resposta, sendo que as respostas corretas totalizam seis pontos, correspondendo a 100%.

Posteriormente, foram aplicados escores adaptados do estudo de Almeida et al.^14^, em que o grau de conhecimento foi categorizado em conhecimento insatisfatório (0 a 74%) e conhecimento satisfatório (75 a 100%). As respostas foram consideradas adequadamente corretas quando: o adolescente discordou que os homens não pegam HPV, concordou que as mulheres vacinadas contra o HPV precisam fazer exames de Papanicolau, concordou que o HPV pode ser contraído por meio de atividade sexual, discordou que o HPV é muito raro, concordou que o HPV pode causar câncer cervical e discordou que a vacina contra o HPV protege contra todos os tipos de câncer.

Quanto à determinação de atitudes, o instrumento foi composto de declarações positivas e negativas sobre os aspectos que envolvem a vacinação e, a cada uma dessas alternativas, atribuiu-se um valor numérico, sendo que a atitude mais positiva totaliza oito pontos, o que corresponde a 100%. Posteriormente, a atitude foi classificada de acordo com o percentual da seguinte forma: de 0 a 74% atitude negativa, de 75 a 100% atitude positiva.

A atitude foi considerada positiva quando o adolescente concordou que valoriza fortemente sua saúde, concordou que a prevenção de doenças e infecções é importante para ele, discordou que ser furado por uma agulha pode incomodar, discordou que teme que as vacinas contra o HPV sejam muito dolorosas, concordou que não tem medo de receber vacinas, discordou que está preocupado com os efeitos colaterais da vacina, discordou que se sente tenso quando ouve outros garotos falando sobre a vacinação contra o HPV, concordou que está preocupado que possa ter câncer provocado pelo HPV no futuro.

A prática foi avaliada por meio do esquema vacinal. Foi considerada prática adequada quando o adolescente respondeu ter recebido as duas doses da vacina contra o HPV.

Todas as variáveis do instrumento para coleta de dados foram organizadas e codificadas em um dicionário denominado de *codebook*. Em seguida, esses dados foram digitados em planilha do Microsoft Office Excel 2016, e após dupla digitação, os dados foram exportados para o *Statistical Package for the Social Sciences* (SPSS), versão 21, para realização das análises estatísticas. Excluíram-se os adolescentes cujos questionários apresentavam incompletude.

Os dados sociodemográficos e a classificação do conhecimento, atitude e prática (situação vacinal) foram analisadas inicialmente, por meio de estatística descritiva. O teste bivariado de associação entre as variáveis qualitativas (sociodemográficas, conhecimento e atitude) utilizado foi a Regressão Logística Simples, referido aqui como *odds* não ajustado, com objetivo de selecionar as possíveis variáveis que poderiam explicar a prática adequada.

Para explicar o efeito conjunto das variáveis sobre o desfecho prática adequada (sim/não) utilizou-se a regressão logística múltipla, por meio da *Odds Ratio* ajustada (ORa). O critério para inclusão de variáveis no modelo logístico foi a associação ao nível de p ˂ 0,20 na análise bivariada^15^. Todavia, o critério de significância das variáveis no modelo foi a associação ao nível de p ˂ 0,05.

Este estudo foi aprovado pelo Comitê de Ética em Pesquisa da Universidade Federal do Piauí, sob o parecer nº 2.868.990, em conformidade com a Resolução nº 466/2012. Os adolescentes assinaram um termo de assentimento livre e esclarecido e os pais ou responsáveis um termo de consentimento livre e esclarecido como forma de comum acordo em participar da pesquisa.

## RESULTADOS

Participaram da pesquisa 472 estudantes, uma vez que, por ocasião da coleta de dados, em uma das salas de aula selecionadas, todos os adolescentes com 15 anos manifestaram o desejo de participar. Do total de adolescentes, 60,8% eram do sexo feminino, 39,8% do sexo masculino e a maioria se definiu como pardos (60,8%). A avaliação do fator econômico resultou em variáveis dicotômicas, tendo como ponto de corte os quintis, em que o grupo com menor posse de bens pontuou até o 1º quintil e o grupo com maior posse de bens pontuou acima do 1º quintil. A maioria dos adolescentes (79,7%) foi categorizada como pertencente ao grupo com maior posse de bens (Tabela 1).

**Tabela 1:** Características sociodemográficas dos adolescentes do estudo. Teresina, PI, Brasil, 2019 (n = 472).

Variáveis	n	%
Sexo		
Masculino	185	39,2
Feminino	287	60,8
Etnia		
Branca	68	14,4
Preta	65	13,8
Parda	287	60,8
Outras (Amarela ou Indígena)	52	11,0
Indicador de bens		
Grupo com menor posse de bens (até o 1º quintil)	96	20,3
Grupo com maior posse de bens (acima do 1º quintil)	376	79,7

O percentual de adolescentes com conhecimento insuficiente sobre o HPV foi de 72,7%, e 65,9% apresentaram atitudes negativas em relação à prevenção contra a infecção. A prática adequada (estar vacinado) foi identificada em 74,6% dos adolescentes (Figura).

**Figura:** Conhecimentos, atitudes e práticas da amostra do estudo sobre o HPV. Teresina, PI, Brasil, 2019.

O sexo feminino encontra-se mais vacinado (92,7%) do que o masculino (46,5%). A variável raça/cor não apresentou diferenças significativas entre as categorias em relação à prática. A avaliação da variável indicador de bens identificou um percentual um pouco mais elevado de adolescentes vacinados (77,1%) no grupo com menor posse de bens (Tabela 2).

**Tabela 2:** Associação entre as variáveis sociodemográficas, conhecimento e atitude com prática de vacinação contra o HPV. Teresina, PI, Brasil, 2019.

Variáveis	Prática (vacinado contra o vírus HPV)		Odds bruta (IC_95%_)	p

	Sim	Não		
Sexo				
Feminino	266 (92,7%)	21 (7,3%)	14,58 (8,58–24,77)	< 0,001
Masculino	86 (46,5%)	99 (53,5%)	1	
Raça/cor				
Branca	52 (76,5%)	16 (23,5%)	0,97 (0,41–2,29)	0,954
Preta	46 (70,8%)	19 (29,2%)	0,72 (0,31–1,67)	0,454
Parda	214 (74,6%)	73 (25,4%)	0,87 (0,43–1,76)	0,718
Amarela ou indígena	40 (76,9%)	12 (23,1%)	1	
Indicador de bens				
Grupo com menor posse de bens (até o 1º quintil)	74 (77,1%)	22 (22,9%)	1,18 (0,69–2,01)	0,527
Grupo com menor posse de bens (acima do 1º quintil)	278 (73,9%)	98 (26,1%)	1	
Conhecimento				
Suficiente	108 (83,7%)	21 (16,3%)	2,08 (1,23–3,51)	0,005
Insuficiente	244 (71,1%)	99 (28,9%)	1	
Atitude				
Positiva	128 (79,5%)	33 (20,5%)	1,50 (0,95–2,37)	0,077
Negativa	224 (72,0%)	87 (28,0%)	1	

ORb: *Odds Ratio* bruta; IC95%: Intervalo de confiança de 95%; p-valor: valor de p.

Ao testar a associação entre as variáveis sociodemográficas, o conhecimento e a atitude com a prática, observou-se que o sexo feminino (ORb = 14,58; IC95%: 8,58–24,77) e o conhecimento (ORb = 2,08; IC95% = 1,23–3,51) foram estatisticamente associados (Tabela 2).

No modelo multivariado, as variáveis que explicaram a prática adequada foram o sexo, o conhecimento e a atitude. Adolescentes do sexo feminino têm 15,62 vezes mais chances de serem vacinados (ORa = 15,62; IC95% = 9,08–26,90), o conhecimento suficiente aumenta 2,09 vezes essa chance (ORa = 2,09 IC95% = 1,15–3,81) e a atitude positiva aumenta 1,89 vezes as chances de vacinação (ORa = 1,89; IC95% = 1,10–3,23) (Tabela 3).

**Tabela 3:** Regressão logística das variáveis associadas à prática adequada contra o HPV. Teresina, PI, Brasil, 2019.

Variáveis	Vacinado contra o vírus HPV		ORb (IC_95%_)	p	ORa (IC_95%_)	p

	Sim n (%)	Não n (%)				
Sexo						
Feminino	266 (92,7%)	21 (7,3%)	14,58 (8,58–24,77)	< 0,001	15,62 (9,08–26,90)	< 0,001
Masculino	86 (46,5%)	99 (53,5%)	1		1	
Conhecimento						
Suficiente	108 (83,7%)	21 (16,3%)	2,08(1,23–3,51)	0,005	2,09 (1,15–3,81)	0,015
Insuficiente	244 (71,1%)	99 (28,9%)	1		1	
Atitude						
Positiva	128 (79,5%)	33 (20,5%)	1,50 (0,95–2,37)	0,077	1,89 (1,10–3,23)	0,019
Negativa	224 (72,0%)	87 (28,0%)	1		1	

ORb: *Odds Ratio* bruta; IC95%: Intervalo de confiança de 95%; p-valor: valor de p.ORa: *Odds Ratio* ajustada.

## DISCUSSÃO

A maioria dos adolescentes participantes deste estudo é do sexo feminino, se autodeclara como pardos e se enquadra no grupo com maior posse de bens. Os dados do perfil dessa amostra referentes a sexo e raça/cor se aproximam da análise da Pesquisa Nacional de Saúde Escolar 2015, realizada com uma amostragem nacional de 102.301 alunos do 9º ano, em que a maioria, 51,3%, pertence ao sexo feminino, e se caracterizou como pardos 43,1%^16^. Estudo realizado no Rio de Janeiro, Niterói e São Gonçalo, em 2018, com 807 adolescentes escolares também enquadrou a maioria dos participantes (80,3%) no grupo com maior posse de bens, embora esse estudo abrangesse alunos de escolas públicas e privadas^12^.

Apesar de o conhecimento sobre o HPV desempenhar um papel muito importante na forma como as pessoas assumem sua sexualidade, proteção e prevenção^17^, os resultados desta pesquisa evidenciaram que a maioria dos adolescentes apresentou conhecimento insuficiente a respeito desse agravo. Corroborando outras publicações que apontam para um conhecimento insuficiente sobre o HPV entre o público adolescente^18^ e em diferentes grupos populacionais^19^. No Brasil, o estudo com amostragem nacional^5^ realizado com jovens de 16 a 25 anos, identificou uma porcentagem média de 51,79% de respostas corretas sobre o HPV e a vacinação. No México^17^, realizou-se uma pesquisa com 242 estudantes com idade entre 14 e 18 anos em que 80% apresentaram baixo nível de conhecimento sobre o HPV.

Observa-se que o público feminino apresentou um nível de conhecimento superior ao masculino, resultado semelhante ao encontrado em outras pesquisas^19,20^. No entanto, ao considerar o nível de conhecimento apenas entre o público feminino, foi evidenciado um elevado percentual (70,7%) com conhecimento insuficiente, como em outro estudo nacional^19^, em que apesar de o grupo de mulheres ter apresentado um percentual superior ao de homens, ainda se percebe uma baixa proporção de indivíduos bem informados.

A maioria dos adolescentes apresentou atitudes negativas em relação à prevenção do HPV e à vacinação. Foram avaliados aspectos relacionados à percepção da importância da prevenção e ao medo e desconforto ao receber a vacina. A percepção da gravidade e da suscetibilidade do indivíduo em relação à infecção pelo HPV, bem como suas crenças sobre os benefícios e barreiras à vacinação podem prever a intenção em receber a vacina, e essa intenção prediz o comportamento^21^.

As atitudes e crenças dos pais e adolescentes em relação à segurança e eficácia da vacina, preocupações acerca dos efeitos colaterais, falta de convicção de que a vacina é essencial, especialmente entre o sexo masculino, além da falta de conscientização sobre a infecção pelo HPV e seus riscos associados, podem ser caracterizados como fatores que dificultam a manutenção de coberturas vacinais elevadas^22^.

No início da implantação da vacina quadrivalente contra o HPV no Sistema Único de Saúde (SUS), que ocorreu em 2014, o país atingiu uma elevada cobertura vacinal na primeira fase da campanha, no entanto, houve uma redução significativa e progressiva para a segunda dose^23^que resultou em um percentual de apenas 60,15% de adolescentes vacinadas^24^. Supõe-se que essa redução tenha ocorrido porque a primeira dose foi administrada nas escolas públicas e privadas, ao passo que a segunda dose ficou disponível nas Unidades Básicas de Saúde, como ocorre atualmente, e poucos profissionais de saúde se dirigiram até o público-alvo para realizar a segunda dose nas escolas^23^. No ano subsequente à disponibilização da vacina houve uma diminuição substancial na cobertura^25^. Esse quadro mostra que a estratégia inicial de vacinação nas escolas adotada no Brasil apresentou mais sucesso nas taxas de adesão.

Os resultados demonstraram que o sexo feminino apresentou chances muito superiores de se vacinar quando comparado ao sexo masculino. O fato de a vacina ter sido disponibilizada pelo SUS inicialmente apenas para o sexo feminino e a difusão de informações relacionadas às consequências do HPV nas mulheres, como o câncer cervical, possivelmente contribui para o desconhecimento da disponibilidade dessa vacinação para ambos os sexos e para as menores coberturas vacinais entre os adolescentes masculinos^26^.

Neste estudo, nenhuma característica sociodemográfica foi associada ao conhecimento e à atitude sobre o HPV. Contudo, em outra pesquisa realizada no Brasil, a raça negra foi associada a baixos níveis de conhecimento sobre o HPV entre as mulheres^27^. Nos Estados Unidos, um estudo evidenciou que as mulheres de raça negra possuíam menor probabilidade de ter conhecimento sobre a vacina do HPV em comparação com as brancas^28^. É provável que esse cenário ocorra devido a um maior acesso à vacina e ao conhecimento sobre o tema entre as pessoas da raça branca, influenciado por fatores socioeconômicos^29^.

O sexo feminino, o conhecimento suficiente e as atitudes positivas sobre a vacina e o HPV foram associados à prática adequada de prevenção por meio da vacinação. Todavia, o baixo índice de adolescentes com conhecimento suficiente e com atitudes positivas representa um cenário preocupante, pois influencia negativamente na aceitabilidade e adesão à vacina, como demonstrado neste estudo e como citado em outras publicações^29,30^, a título de exemplo, a realizada com estudantes do ensino médio na Malásia^30^, a qual identificou que a intenção de se vacinar foi significativamente associada ao nível de conhecimento sobre o câncer do colo do útero. Tal cenário evidencia a necessidade de intervenções educativas voltadas para a conscientização da população a respeito da problemática da infecção e da necessidade de adoção de medidas preventivas.

A principal força deste estudo foi realizar uma investigação com uma amostragem que abrangeu todas as escolas públicas da zona urbana do município de Teresina, o que forneceu um panorama sobre os conhecimentos e atitudes desses estudantes em relação ao tema e permitiu uma avaliação das taxas de adesão à vacina contra o HPV. Outro ponto forte desta pesquisa foi abranger adolescentes que recentemente fizeram parte do público-alvo da imunização contra o HPV na rede pública de saúde.

Dentre as limitações, pode ser citada a inexistência de uma escala padronizada para avaliação de conhecimentos e atitudes a respeito do tema, a opção pelo procedimento de pontuação ocorreu por acharmos mais adequado do que avaliar com perguntas isoladas. No entanto, considera-se que esta investigação poderá fornecer importantes contribuições para as estratégias e políticas de saúde voltadas para a intensificação da cobertura vacinal contra o HPV. A ocorrência de vieses de informação, também é possível considerando-se as respostas autodeclaradas, sobretudo aquelas relacionadas às questões sexuais, que poderiam levar os adolescentes a alterarem as suas respostas por receio de julgamentos morais. Para minimizá-los foram utilizados instrumentos com autorresposta.

## CONCLUSÃO

Os achados deste estudo demonstraram associações significativas entre o conhecimento suficiente e atitudes positivas com a prática da vacinação, o que evidencia que ampliar o conhecimento dos adolescentes, gerando atitudes favoráveis, pode ser uma importante ferramenta para adesão à vacina contra o HPV.

Os resultados também evidenciaram um baixo índice de adolescentes masculinos vacinados e uma associação entre o sexo feminino e a vacinação contra o HPV. Esse cenário indica a necessidade de incluir a população masculina em temas inerentes à prevenção ao HPV, pois frequentemente a infecção é associada apenas ao câncer do colo do útero, subestimando-se as suas consequências entre os homens, o que contribui para as baixas coberturas vacinais nesse público.
